# The value of monitoring data in a process evaluation of hygiene behaviour change in Community Health Clubs to explain findings from a cluster-randomised controlled trial in Rwanda

**DOI:** 10.1186/s12889-019-7991-7

**Published:** 2020-01-23

**Authors:** Juliet Waterkeyn, Anthony Waterkeyn, Fausca Uwingabire, Julia Pantoglou, Amans Ntakarutimana, Marcie Mbirira, Joseph Katabarwa, Zachary Bigirimana, Sandy Cairncross, Richard Carter

**Affiliations:** 1 Africa AHEAD–UK, 95 Dorries Drive, Simon’s Town, South Africa; 2 Africa AHEAD-UK, 5 River Road, Littlehampton, UK; 3 University of Kibungo, Ngoma, Rwanda; 4 Institute of Tropical Medicine and International Health, Berlin, Germany; 5 College of Medicine and Health Sciences, University of Rwanda, Kigali, Rwanda; 6 Africa AHEAD-Rwanda, Kicukiro, Kigali, Rwanda; 7 London School of Hygiene and Tropical Medicine, Keppel St, London, WC1E 7HT UK; 8 Richard Carter and Associates Ltd, The Oxlip, Amphill, MK45 2EH UK

**Keywords:** Hygiene behaviour change, Community Health Clubs, Rwanda, Randomised controlled trial, Health impact

## Abstract

**Background:**

A cluster-Randomised Controlled Trial evaluation of the impact of the Community Health Clubs (CHCs) in the Community Based Environmental Health Promotion Programme in Rwanda in 2015 appeared to find little uptake of 7 hygiene indicators 1 year after the end of the intervention, and low impact on prevention of diarrhoea and stunting.

**Methods:**

Monitoring data was revisited through detailed community records with all the *expected inputs*, *outputs* and *external determinants* analysed for fidelity to the research protocol. Five household inventory observations were taken over a 40-month period including 2 years after the end of the cRCT in a random selection of the 50 intervention CHCs and data compared to that of the trial. Focus Group Discussion with all Environmental Health Officers of the Ministry of Health provided context to understand the long-term community dynamics of hygiene behaviour change.

**Results:**

It was found that the intervention had been jeopardised by external determinants with only 54% fidelity to protocol. By the end of the designated intervention period in June 2014, the treatment had reached only 58% of households with 41% *average attendance* at training sessions by the 4056 registered members and 51% *mean completion rate* of 20+ sessions. Therefore only 10% of 50 CHCs provided the full so-called ‘Classic’ training as per-protocol. However, sustainability of the CHCs was high, with all 50 being active 2 years after the end of the cRCT and over 80% uptake of recommended practices of the same 7 key indicators as the trial was achieved by 2017.

**Conclusions:**

The cRCT conclusion that the case study of Rusizi District does not encourage the use of the CHC model for scaling up, raises concerns over the possible misrepresentation of the potential of the holistic CHC model to achieve health impact in a more realistic time frame. It also questions the appropriateness of apparently rigorous quantitative research, such as the cluster-Randomised Controlled Trial as conducted in Rusizi District, to adequately assess community dynamics in complex interventions.

## Introduction

For more than three decades the literature has been clear that diarrhoeal disease can be reduced by well-conducted health promotion activities [1, 2] and that if handwashing with soap is done at critical times, diarrhoea can be reduced very significantly [3]. In 2010, there were 17,319 reported cases of diarrhoea accounting for 23% of all outpatient visits of under 5’s at Health Centres in Rwanda [4]. As CHCs in Zimbabwe had previously demonstrated significantly changed hygiene behaviour within 1 year [5], this model was chosen by the Rwandan Ministry of Health (MoH) in the hope that Community Health Clubs (CHCs) in every village would provide a means to change hygiene behaviour and reduce diarrhoea. The CHC model was adopted as the mechanism for behaviour change for the national Community Based Environmental Health Promotion Programme (CBEHPP). In the Health Sector Strategic Plan (HSSP III) [6], the government reiterated the use of *‘Community Health Clubs with enhanced health promotion and behaviour change capacity’* with the target of reaching 70% of all villages in Rwanda by 2018 with all implementing agencies conforming to this national strategy. By 2015, 90% (13,472) of villages throughout all 30 Districts had registered CHC members, of which 40% (5433), had also provided training in health promotion using the standard manual [7] usually with the support of Non-Governmental Organisations (NGOs). Community management of hygiene and sanitation improvements were implemented by MoH through the CBEHPP, but progress was carefully monitored through a uniquely Rwandan system known as *Imihigo*, in which every District Mayor is held responsible for meeting certain development targets (including the number of CHCs in the District) through a personal contract with the President.

## Background

### The Community Health Club theory of change

The Community Health Club strategy is based on a theoretical model which identifies a series of stages of change. Progress can be thwarted on two levels: at macro (national) level, if there is a lack of leadership through government, and at micro level if there is a lack of community compliance at village and district level. Health impact is contingent on a critical mass of households voluntarily introducing changes to their hygiene practices (Table 1).

**Table 1:** The Theory of Change for a Classic Community Health Club Intervention – as used in Rusizi District, Rwanda

Causes (Determinants)	Effect	Results	Expected inputs (Assumptions)	Outputs	Outcomes
MACRO LEVEL: National / Provincial and District					
1.1.i. Fragile State -breakdown of economy, law, order and security	ii. Government structures are weakened or ineffectual	iii. Emergency humanitarian programmes take over from normal state structures	iv. Political enabling environment. Government (MoH) provides normal services /support	v. Political enabling environment: NGOs / funding agencies support national government CBEHP program	vi. Funding: NGOs and Agencies are able to provide financial and advisory support to districts
1.2.i. Lack of a clear Environmental Health (EH) Strategy within MoH policy / government reshuffle or changes in administration	ii. Environmental Health Department (EHD) is weak and doesn’t manage the WASH sector	iii. Uncoordinated WASH sector /many different strategies and conflicting models of change	iv. EH Policy: Development of a national Road Map for CBEHPP using CHCs in each village with clear methods to achieve behaviour change	v. Higher political visibility - EHD manages the CBEHPP with support for MoH by donor agencies and NGOs	vi. WASH programs can be scaled up and CHC started throughout country
1.3.i. Lack of standardised training materials	ii. Difficult to train trainers effectively	iii. No Core -Trainer of trainers team	iv. Training Material: Develop CBEHPP manual and tools to be readily available	v. National Core Trainers trained in CBEHPP to train all districts at every level	vi. Sustainable human resource in country to implement CBEHPP
1.4.i. Lack of WASH strategy in District	ii. Weak budgetary support & inadequate training for EHOs	iii. District prioritises curative over preventative EHD services	iv. Training Trainers: EHOs and district leadership understand the rationale for starting CHC	v. EHOs monitor CHC and have to account for progress on WASH indicators in CBEHPP	vi. Sustainable district planning and monitoring systems ensuring CHCs continue to function
1.5.i. Lack of transport for EHOs to monitor CHCs	ii. Community monitoring does not take place	iii. Little data on hygiene/ sanitation in villages	iv. Transport: EHOs are provided with reliable motorbikes to reach villages so as to monitor CHCs	v. Mobile EHOs are able to monitor CHCs easily	vi. SDG WASH targets are tracked and therefore more likely to be met at district level
1.6.i. Low profile of EHD in Districts	ii. Not enough EH staff in district	iii. Inability of MoH to properly monitor WASH	iv. Supervision: EHOs supervise CHC facilitators in community	v. CHC facilitators well supported in ensuring active and effective CHC	vi. High Profile of EHD in district
1.7.i. Lack of Meeting venue	ii. Difficult to hold CHC sessions in heavy rainy season	iii. Low CHC attendance due to meeting held outside in rain	iv. Timing / Duration: 24 CHC health sessions have to be timed to be held in the dry season	v. High Completion of training – no excuse for members not to complete training	vi. High coverage of well informed CHC members and active group in all villages
MICRO LEVEL: Village and household					
2.1.i. Poorly organised community	ii. Low levels of hygiene and sanitation	iii. High diarrhoea rates and resistence to change	iv. Community Mobilisation: A CHC is started in every village	v. Peer support for all households to change with social pressure to meet hygiene standards	vi. ^a^80% housholds are in a CHC sharing same attitudes, beliefs, values.
2.2.i. Lack of informed leadership	ii. Poor decision making	iii. Lack of training and monitoring of hygiene standards	iv. Quality Training: CHC facilitators / leaders are trained in participatory CHC approach & CBEHPP	v. CHC Facilitator within village / village leaders trained to monitor hygiene standards	vi. ^a^50-100 households are active members within a functional CHC
2.3.i. Lack of learning opportunity within village	ii. Inadequate knowledge to prevent disease	iii. Little community action to improve WASH facilities	iv. Exposure: 24 CHC health sessions are offered weekly for at least 6 to 12 months	v. Improved understanding how to prevent disease by safe hygiene and sanitation	vi. ^a^80% of households with knowledge of how to manage family health
2.4.i. Inertia and lack of interest in hygiene & prevention of disease	ii. Not prioritising ways to protect their family	iii. Poor hygiene & little effort/ expenditure on improving WASH facilities	iv. Visibility: Model Home competitions are held to increase interest & attract high level of participation	v. High priority in the investment of time and energy to improve hygiene facilities and change behaviour	vi. ^a^80% uptake of safe hygiene practice and safe sanitation facilities
2.5.i. High risk hygiene practices and sanitation	ii. High levels of preventable disease	iii. High infant and child mortality	iv. Reinforcement: CHC continue to meet after the CHC training is complete	v. Higher social cohesion and increased support for vulnerable individuals	vi. Improved social capital, family health^b^ and standard of living.

^a^The target of intervention varies depending on the intervention design – This table shows the CBEHPP target in Rwanda. Over 80% compliance of recommended practices (safe drinking source, safe water storage, safe sanitation, zero open defecation, hand washing facility, soap for handwashing, pot racks /clean pots, solid waste managed, individual cups/plates, safe food hygiene, dedicated clean kitchen, grey water drainage

^b^For the Stage 1 Training in CHC which focuses on WASH mainly a decrease in diarrhoea, skin disease, bilharzia, intestinal parasites is possible

At micro level, the CHC model can reasonably predict that if the *expected inputs* (quantity and quality of training) are provided as per-protocol, and if individual CHC members participate in at least 24 health promotion sessions (one per week for six consecutive months), with at least 1 year follow-up to reinforce non-risk practices, peer pressure from other CHC members, combined with the understanding of disease prevention, is likely to result in the improved hygiene behaviour of the CHC member [8]. As a critical mass of the population of the village should be compliant if any health impact as a result of the CHC training is to be achieved, the target of the intervention is to enrol at least 80% of the households within a village into a CHC, and to achieve at least 80% of that membership to adopt all the key recommended hygiene practices [5] before any impact on diarrhoea can reasonably be expected. Households which do not have a member which actively participate in the CHC training are not as likely to change their hygiene behaviour merely by virtue of the trickle-down effect of living in a village where there is an active CHC although some may emulate without full comprehension of the reasons for such change [9].

### The intervention

In 2013, Rusizi District was selected to evaluate the impact of the CHC model, being one of the most remote districts of Rwanda, with a relatively high WASH-attributable disease burden at the time. No material resources were to be provided to the community in the intervention to upgrade water and sanitation facilities, nor was there to be any practical agricultural component in the training. The sole input of the intervention was providing everyone in each village the opportunity of joining a Community Health Club, enabling CHC Members to attend 24 two-hour free health promotion sessions over a 6 month period, thereby increasing their health knowledge through participatory group activities based on the national CBEHPP Manual [7]. The training was to be co-ordinated by a Village Health Worker (VHW) and supervised by an Environmental Health Officer (EHO) from the Ministry of Health. No refreshments were provided at the training, nor were T-shirts given to CHC members: the only incentive to attend sessions being a psychological sense of personal achievement and the honour of being awarded a certificate in a public ‘graduation’ ceremony for those who completed more than 20 topics.

A unique feature of the CHC model is the use of a Membership Card to mobilise the community to join the club, and to enable planning and monitoring attendance at village level [8]. Unlike many methodologies for mobilising community which attract a loose gathering of villagers, the CHC Model registers a defined membership, so that the exact number reached by the training is known, and progress can be made with the same members building on their knowledge from week to week. It is a core practice of the model that each member *must* have a Membership Card (Table 2) which is kept at home and presented, at each session they attend, to the CHC facilitator who must sign off each topic which is attended, along with the date. This community monitoring system provides a fairly accurate record of each member’s attendance, especially when double checked against the CHC attendance register held by the facilitator. Membership cards are also used for spot checks by the EHO supervisors to determine which topics have been completed by the CHC and to check that only one ‘health topic’ is being undertaken per weekly session. In an ideal scenario, the EHO’s responsibility is to assist in some of the more complex topics, and to ensure the training is not being rushed through in less than the mandatory six-month period and that participatory activities are used by the CHC facilitator.

**Table 2:** Membership Card with 24 topics, showing the topics to be covered and the recommended practices as developed in the CBEHPP by Ministry of Health Rwanda

	TOPIC	DATE	SIGNATURE	RECOMMENDED PRACTICES	DATE COMPLETED
1	What is a CHC			Registration of neighbours	
2	Electing a committee			Vote for a committee	
3	Mapping			Village map with all WASH facilities	
4	Household Inventory			Take part in a home survey	
5	Personal hygiene			Washing clothes, blankets	
6	Skin/eye diseases			Treatment of skin /eye diseases	
7	Handwashing methods			Used hand wash facility	
8	Infant care			Correct child Immunisation	
9	Diarrhoea			Oral Rehydration Solution demo	
10	Malnutrition			Growth monitoring card	
11	Food storage			Pot rack, hanging baskets	
12	Kitchen hygiene			Smokeless stove/ventilated kitchen	
13	Food variety			home nutrition mounds/gardens	
14	A balanced diet			Individual plates and shelves	
15	Water storage			Covered, treated water	
16	Drinking water			Drinking ladle, individual cups	
17	Water Sources			Use of safe drinking water source	
18	WASH management			Clean up of water source	
19	Vector control			Solid waste management/pit	
20	Safe sanitation			Zero Open Defecation (ZOD)	
21	Sanitation planning			Improve/build new latrine	
22	Model Home competition			Home visits: social networks	
23	Drama and songs			Competitions: learn song/slogan	
24	Graduation			Celebrations: attendance	

### The cluster-randomised controlled trial

An external evaluation in Rusizi, one of 30 districts in Rwanda, was carried out in 150 villages by Innovations for Poverty Action (IPA) (2013–2015) using a cluster-Randomised Controlled Trial (cRCT) design. Villages were allocated to each of the three research arms, of which 3616 households were assigned to the control, 3196 to the Lite arm (with 8 sessions of training) and 3464 to the Classic Intervention (which would receive the full 24-weeks of training as per the CBEHPP Manual). The IPA team enrolled 8734 households with children under 5 years, with a base-line survey in 2013 which was compared with 7934 (91%) of the same households in an end-line survey in 2015. The main treatment variable was the intervention status of the village where the individual lived at baseline, not whether the household was enrolled in a Community Health Club. The primary outcome was caregiver-reported diarrhoea in children under 5 years within the previous 7 days. To ascertain hygiene behaviour change in CHC villages, seven standardised indicators were employed by IPA in the cRCT: *(i) safe water source, (ii) water treatment, (iii) improved sanitation facility, (iv) structurally complete sanitation facility, (v) faeces seen in yards, (vi) sanitary disposal of children’s faeces, (vii) handwashing station with soap and water* [10].

## Methods

Conducting a process evaluation alongside or as part of experimental trials is normally considered good practice [11, 12]. As this was not provided by the IPA evaluation team, and because we had been responsible for the monitoring component of the same intervention, the main purpose of this study, is to examine whether the training, as it was conducted in the 50 classic intervention villages of Rusizi District, was sufficient to expect a reduction in diarrhoea and stunting and whether contextual factors external to the intervention may have affected the intermediate results of the cRCT.

### Data collection

#### Community response

Each CHC in Rusizi District was obliged to keep a registration book of the number of members, the average attendance per session, the number of sessions provided, and which topics were covered, as well as the number who completed all topics and graduated, as shown on the membership card (Table 2). To enable the ranking of CHCs within the district, membership cards were collected from each CHC 1 year after the end of the training, entered into excel and a database established online through a dedicated CBEHPP website [13] with all the 150 CHCs’ details recorded.

#### Monitoring of hygiene behaviour change

To assess levels of hygiene behaviour change, we compared a series of random surveys in each CHC using the household inventory developed for CBEHPP (see Additional file 1). The ‘household inventory’ was developed during the intervention by the corresponding author through Africa AHEAD for Ministry of Health (MoH/AA) and used as a checklist of 50 empirical observations in each household, avoiding any *reported* claims of safe hygiene behaviour. A series of five surveys, using the household inventory was taken over a 40-month period from October 2013 to February 2017.

- A baseline of all 50 future CHC villages was taken by Village Health Workers in 5745 households (81% of the 7086 households) in October 2013.
- A midline survey (2–3 months into training) was taken in 424 (10.4%) of the 4056 CHC households in 30 out of 50 randomly selected ‘classic’ villages, in April 2014.
- An end-line survey (6 months after training) in 738 (18%) of the same number of households in 24 randomly selected ‘classic CHC’ villages in December 2014.
- A post-intervention survey (22/23 months after the training ended) in 408 (10%) households from all 50 CHCs, with 10 respondents per CHC in May 2016.
- A final survey (33/34 months after the training ended) in 604 (14.8%) households in 24 CHCs purposefully selected if they had been previously surveyed twice, with random sampling of 20 members per CHC in February 2017.

Within each round of data collection, the standard CHC register was used as a sampling frame for each CHC where every *n*th member was selected depending on size of club. Data was collected by EHOs assisted by student teachers who were trained in a one-day workshop, using a mobile application developed especially for monitoring CBEHPP.

#### Context

The intervention was more deeply understood in retrospect by using secondary data: emails, local media, project records and accounts, as well as key informant interviews with field personnel to analyse the *external determinants* - a set of contextual factors that may influence the ‘inputs’ but which cannot be controlled by the implementers.

### Data analysis

- *Community Response* was gleaned from CHC records and membership cards and the data entered into Excel and compared simply by the using mean and mode. The *‘popularity’* of each CHC was analysed by project officers using four main criteria: *Size, Spread, Treatment and Completion* (Table 3) in order to rank the 50 CHCs.
- *Hygiene behaviour change* was analyzed by selecting seven intermediate outcomes from 50 indicators in the monitoring data to match as closely as possible with those used in the cRCT evaluation [10]. All quantitative data from the 5 surveys were analysed in Excel and SPSS using the Pearson Chi squared test for significance (Mantel-Haenzel test for trend) (Table 5).
- *A Process Evaluation* of the intervention as per protocol was conducted in terms of *Scope*, *Choice, Definition of Indicators*, *Methods of Data Collection*, *Timing *and *Intermediate Outcomes* (Table 4).

**Table 3:** Summary of Mobilisation Targets of intervention in 50 Classic CHC

Target	Community Outputs	CHC achieving targets	
	Per-protocol target	*n = 50*	%
1. Target 1: Size	> 70 members are registered in a CHC	32	64%
2. Target 2: Spread	> 80% households of village in a CHC	12	24%
3. Target 3: Treatment	> 50% average attendance per session per CHC	14	28%
4. Target 4: Completion	CHC provide > 24 sessions	25	50%
5. Two targets	CHCs attaining Target 1&2	10	20%
6. Three targets	CHCs attaining Target 1 & 2 & 3	7	14%
7. Four Targets	CHCs attaining Target 1 & 2 & 3 & 4	5	10%

**Table 4:** Basic Assumptions of a Classic CHC project compared to the intervention as performed in Rusizi District (2014–2015)

Basic Assumptions of ‘classic’ (per-protocol) CHC	Score	cRCT intervention as implemented	Score
Training material			
A customized CHC Training Manual	4	CBEHPP Manuals were available and used	4
Training manual developed/approved by MoH	4	Manual available and used	4
A tool kit of culturally appropriate visual aids	4	Appropriate visual aids available/well used	4
Sub total	12		12
Trainers			
Sufficient NGO Project staff to support EHOs	4	Not sufficient - only one dedicated PO for district	2
District leadership to ensure full local support	4	Mayor & District Health Officer removed from post	1
EHOs to mentor CHC Facilitators	4	Only 6 EHOs to supervise CHC facilitators	2
Politically enabling environment	4	Minister & Head MoH disabled CBEHPP	0
The CHC Facilitators are Village Health Workers	4	No public health personnel facilitated CHC	2
All CHC facilitators get a 5-day training	4	High turnover/30% had to be retrained in situ	3
Sub total	24		10
Transport			
EHOs to have motorbikes	4	Motorbikes provided but after the training in Year 2	1
Project staff to have dedicated vehicle	4	No vehicle/motorbikes used on dangerous roads	1
VHWs to have bicycles	4	Supplied but not appropriate as hilly terrain	4
Sub total	12		6
Training			
Size of CHC: at least 70 members	4	32 CHCs (64%) reached > 70 members	3
Coverage: 80% of village HHs in CHC in Y.1.	4	12 CHCs (24%) reached 80% coverage in Y1	2
All CHC sessions are participatory	4	Condensed sessions, so less participatory	3
Only one key message and one homework	4	Many messages and multiple homework	3
Model Home Competitions held end of training	4	Few competitions were held during intervention	0
CHC Membership Cards used / signed	4	CHC membership cards were used and signed	4
Certificates given at Graduation Ceremony	4	Only 50% of CHC held Graduations	2
Club venues permanent demonstration sites	4	Very few venues permanent or had demonstrations	0
Sub total	32		17
Timing			
Training is conducted during the dry season	4	All training conducted in the rainy season	0
Six months continuous weekly training	4	Only 4–5 months available for training	2
24 health sessions meeting once a week	4	Only 4 CHC (8%) held > 20 sessions (mean of 15)	2
2 h for each session provided	4	At least two hours if more than one topic was done	3
Only one topic is done per session	4	On average 2 topics done per session	2
Sub Total	20		9
Total possible Score	100	Total Score	54

The results from both the quantitative and qualitative data were presented in a Focus Group Discussion during the final survey 33 months after the end of the intervention to 28 relevant district stakeholders who had been involved in the intervention including all EHOs and their supervisors. These government officials were divided into two groups, and each group analysed the findings in terms of the *‘Expected Inputs’* i.e. the qualitative and quantitative attributes of the intervention - its resources and timing - which were assessed according to five main criteria comparing the quality of the intervention against the CBEHPP guidelines [6]. Each component was rated on a scale from 0 to 4, with ‘0’ indicating no input and ‘4’ being maximum input (Table 4).

### Limitations

Although the CHC membership data were collected by voluntary CHC Facilitators reporting on their own area, it was supervised and collated by EHOs who triangulated with village records where membership cards were lost or unreadable due to wear and tear. Only one of the 50 CHCs failed to produce membership cards for analysis. The household inventory used to monitor hygiene behaviour change was being developed for wider use in CBEHPP nationally and being adapted for a digital data collection tool. As the survey was being refined at each round, it was a challenge to match the different surveys exactly, which resulted in many cases being lost, especially from the baseline. Although we use project monitoring data for this paper, we have attempted to minimise interviewer bias by having an external researcher verify data through spot observations and cleaning any questionable cases [14, 15]; most of the co-authors are not impartial having been part of the monitoring team, but the data were peer reviewed in a thesis whilst external research advisors have examined our raw data.

## Results

### Community response

The intervention wing of the cRCT required 50 fully functional CHCs as per the Classic CHC model, as outlined in Table 4 and explained more fully below. Monitoring records show that there was little resistance from the community to the idea of forming CHCs except in one area of the main town, which failed to start up as the facilitator and members were fully occupied as traders. However, this does not indicate that CHCs cannot succeed in an urban context, as for example, in another high-density suburb, there was such high membership that the CHC had to split into two CHCs of 93 members each to accommodate the full coverage of the area. Therefore, the target number of 50 CHCs were operational as per-protocol. Full data is available in Additional file 2: Table S1 whilst Table 3 below, summarises the findings of four main indicators used to ascertain level of community response, namely *i) Size, ii) Spread, iii) Treatment and iv) Completion.*

(i) *Size:* The target for ‘Number of Registered Members’ was > 70 members per CHC, and this was achieved in 49 CHCs (98%), with all CHC having more than 30 members. In fact, the mean number of members for all 50 CHCs was 80 members (ranging from 36 to 167) per CHC, which surpassed the target: 72% of CHCs achieved a high membership of 70–100 members, 26% of CHCs had a good membership of 50–69 members.
(ii) *‘Spread: The target* was to include 80% of households within a village given the average size of a village was 137 households. By June 2015 the intervention had reached 4056 (58.4%) of 6866 households in the 50 villages (ranging from 23 to 100% of households in a village). High coverage of over 80% was achieved by only 11 (22%) CHCs, good coverage was achieved by 17 CHCs (33%), average coverage by 16 (31%) and low coverage of under 40% of households in a village by only 7 CHCs (14%). However, what could be considered a low coverage in a CHC intervention is in fact still a considerable achievement comparative to other types of WASH interventions such as PHAST [16] and CLTS [17]. Coverage of over 80% would have been possible (as demonstrated by well mobilised villages) but was too ambitious a target for most CHCs with the constraints of the cRCT, reasons for which will be discussed below.
(iii) *Treatment:* The target for ‘Average Attendance’ was a mean of 50% of members at weekly sessions being the norm based on previous experience [5, 18]. This was slightly under-achieved with a mean of 41% of registered members attending weekly sessions. There was however a wide range of attendance levels, with 15 CHCs (30%) reaching this target of 50%, of which 4 CHCs achieved > 75% attendance. The most common category with 20 CHCS (40%) was ‘moderate attendance’ (30–40%) whilst 15 CHCs (30%) had ‘low attendance’ of < 30% of members.
(iv) *Completion:* To complete the whole training requires an exceptionally high level of personal effort from members as they must attend > 20 topics, one per week for 6 months. This commitment in time and energy is a high demand relative to other similar training models such as PHAST [16] which expects someone to attend only 4–6 participatory dialogue sessions, and Community Led Total Sanitation (CLTS) which requires only 1–2 meetings attended by villagers [17]. The target was to achieve at least 50% of members graduated in the first year. This target was exceeded in 25 CHCs (50%) with a mean of 60% graduation of members of all 50 CHC, of which 6 CHCs (12%) achieved a high level of 80–100% graduates. Unlike a regular CHC programme where sessions can continue until the community has been properly served, the cut-off date imposed by the cRCT trial protocol meant there was no opportunity to complete the training nor was it possible to repeat topics on demand.

From these project records we deduce that the CHC sessions were at an appropriate level for the largely semi-literate Rwandan communities who voted with their feet to attend sessions, in a personal effort to increase their knowledge, despite the inconvenience of the poor weather. The gender of facilitator did not affect attendance levels as there was no pattern of high performing CHCs having either men or women as facilitators.

*A Classic CHC:* Whilst the four targets, when taken *individually,* give some indication of community response, according to the CHC Theory of Change *all four targets* (Table 3) should be met in each CHC before there is a critical mass with enough spread of safe hygiene and sanitation to impact on preventable disease. Despite immense efforts in the field, only 5 (10%) out of 50 CHCs met *all four* targets within the 5 months of the intervention. We conclude therefore, that the intervention at the time it was evaluated by the cRCT, did not in fact qualify to be considered a ‘classic’ CHC intervention as per-protocol.

### Comparative results of hygiene behaviour change between cRCT and MoH/AA data

#### Unit of measurement

The cRCT’s unit of measurement is the village as a whole - the independent variable being the household’s situation in a Control village, a Lite village or a Classic village, with a random sample taken of *all* the households in that village (whether they were in a CHC or not). The reason that the results may vary between the cRCT and MoH/AA data is that the two different units of measurement are not strictly comparable. The MoH/AA data randomly sampled the *treated* households (i.e. the Community Health Club, not the whole village); the independent variable was the number of sessions attended by a CHC member as shown on their membership card. We question how the cRCT’s random sample of *all* households in a village could be used to measure the effectiveness of CHC *treatment* in reducing diarrhoea when only 11 out of 50 Classic villages were properly treated.

#### Indicators

Of the 7 indicators used by the cRCT, we consider that two indicators (*latrine ownership and zero open defecation - ZOD*) were poorly chosen as high levels (over 90%) were already evident at the baseline. The two water indicators (*water source and water treatment*) may have been confounded by external programmes taking place in some, but not all, of the same areas: *water source* by provision of municipal piped water throughout Rusizi during the intervention period; *water treatment* due to a programme of wide distribution of water filters. The two sanitation indicators (*improved sanitation facility* and *structurally complete sanitation facility*) used standard WHO definitions which were not adapted to match the targets of CBEHPP. This leaves only one indicator *hand-washing station with soap and water* as an appropriate measurement of hygiene behaviour change. However, as the cRCT bundled together the three components of a safe handwashing facility (station, soap and water) their results for safe handwashing may not indicate the true picture. With that caveat we provide the following comparison between cRCT and MoH/AA data (Table 5).

**Table 5:** Intermediate outcomes in 50 Classic villages in Rusizi District over 40 months as monitored by Ministry of Health/Africa AHEAD (2013–2017)

Survey Type	Base Line	Midline	End Line	Post Intervention	Final	Significance
Research Arm	ALL	CLASSIC	CLASSIC	CLASSIC	CLASSIC	
Data collection period	Oct-Nov	April–May	Dec	April–May	Feb-Mar	
Year of data collection	2013	2014	2014	2016	2017	
Number of CHC sessions attended	None	8–13	19+	19+	19+	
	*n* = 5745	*n* = 738	*n* = 424	*n* = 407	*n* = 644	*p* value ^a^
Drinking water from improved source	3.455 (60%)	493 (67%)	292 (69%)	301 (74%)	471 (73%)	< 0.0001
Adequate drinking water treatment	2.131 (37%)	398 (53%)	367 (87%)	341 (91%)	562 (89%)	< 0.0001
Improved Sanitation^b^	3.816 (67%)	40 (5%)	51 (12%)	285 (71%)	528 (83%)	< 0.0001
Household ownership of a latrine	5.089 (89%)	676 (92%)	406 (96%)	392 (97%)	595 (94%)	< 0.0001
Zero Open Defecation (ZOD)^c^	5.622 (98%)	723 (98%)	421 (99%)	407 (100%)	644 (99%)	< 0.0001
Handwashing facility (tippy tap) ^d^	539 (9%)	107 (15%)	321 (76%)	249 (61%)	–	< 0.0001
Soap available for handwashing^e^	2.498 (44%)	378 (87%)	364 (87%)	407 (99%)	644 (99%)	< 0.0001

^a^Mantel-Haenzel test for trend

^b^Pit latrines with a sealed cover

^c^Sanitary disposal of child feces/feces not visible in courtyard

^d^Due to an oversight hand washing facilities were not monitored in the final survey

^e^Soap can be kept in the household, not necessarily at the tippy tap

(i) *Safe Water:* The cRCT data shows a 4% increase in all the three research arms in use of a *‘safe water source’* by 2017 and we surmise this is probably due to the new piped water scheme. The effect of CHC training may show slightly in ‘*treatment of drinking water’* which increased by 4% more in Classic villages than Lite and Control whilst cRCT per-protocol analysis showed well-trained CHC households (those which had attended > 20 sessions) treated drinking water 9% more than CHC households which had attended only a few sessions. MoH/AA monitoring data found a highly significant response (*p* < 0.001) for water treatment by December 2014 with a 50% increase (37 to 87%) before and after training which rose to 91%, before dropping slightly to 89% by 2017 (Table 5). It is surmised that the distribution of water filters could have fuelled this uptake, as although the new technology was freely available to all households, it would be more likely that CHC members would take advantage of the opportunity.
(ii) *Sanitation:* Although both sets of findings agree that around 90% of households already had access to a latrine of some sort, either at their own home or shared with a neighbour, there is confusion over exact definitions. The cRCT data show only 5% of households with *‘structurally complete sanitation facilities’* at baseline, increasing to 35% at end line. As this one indicator includes many components (floor, walls, roof etc.) it is difficult to unpack. MoH/AA data simply noted *ownership* of any type of latrine (no sharing) and found that household latrine ownership rose from 89 to 94% during the 34 months after the intervention ended. For *‘improved sanitation’* both the cRCT data (66%) and MoH/AA data (67%) concur. However, the cRCT data shows this indicator unaccountably falls to 44% in classic CHC villages. It is important to know that the MoH/AA data definition of a ‘structurally complete latrine’ in Rusizi was changed after the baseline to include a well-sealed fly-proof cover over the pit latrine squat hole. With this additional requirement, *‘improved sanitation’* immediately reflected only 5% compliance at baseline, but with time saw a gradual improvement to reach 83% of CHC households with improved sanitation (including a covered squat hole) by the final survey. The cRCT reported that only 15% of households had ‘*faeces seen in yards’* at baseline which decreased to 9% at end line - a positive outcome - but at the same time they found that the *‘sanitary disposal of child faeces’* in a latrine also fell from 90 to 66% - a negative change. Logically, one would expect that if the *‘sanitary disposal of faeces’* fell, then *more* toddler faeces would be seen in the yard. MoH/AA monitoring records showed that *open defecation* was almost non-existent in the baseline (2%) and fell to almost zero in CHC households’ yards by the final survey. The cRCT failed to report this specific indicator at the end of the intervention.
(iii) *Handwashing:* The cRCT data found that *‘hand-washing facilities with soap and water*’ showed little difference between the three arms, and only 2% difference between those CHC who had attended all 20 sessions and those with one session of health promotion. By contrast, the MoH/AA monitoring data showed that whilst only 9% of CHC members had a tippy tap (a home-made facility for hand-washing) at baseline, 61% of CHC members had one or more tippy taps in the compound 2 years after the intervention had ended (*p* < 0.001) – a very strong response compared to the negative finding of the cRCT 1 year after the end of the 5 month training. MoH/AA data showed 44% of households used soap or ash for handwashing at baseline and this rose to a sustained level of 99% compliance, 34 months after the end of the intervention (Table 5). As the trial found virtually no evidence of ‘*hand-washing with water* *and* *soap*’ 1 year after the intervention ended, we suggest that by bundling of *soap* with the hand-washing station, this finding may have been skewed. We consider that the construction and maintenance of a hand-washing facilities is an achievement of itself, even if soap is not present, as it is the start of a process of change. Interestingly, more local context was revealed in Focus Group Discussion as practitioners in Rusizi explained that as soap is a scarce commodity, households normally would keep their soap in the house, not outside on the hand washing station where it can be stolen, wasted by children, eaten by goats, birds or rodents - an example of contextual nuance which was left unexplained in the cRCT data. Thus, lack of soap at the handwashing facility led the cRCT to give the mistaken impression that the CHC training had virtually no effect on handwashing in the cRCT data. By contrast the MoH/AA method of assessing the use of soap for handwashing was to ask a child to *demonstrate* how they washed their hands and to observe if they fetched the soap from the house to wash their hands.

### Context

The indicators which would affect *community response* in terms of *i) ‘size of village’, ii) ‘duration of training period’, iii) ‘number of meetings provided,* and *iv) ‘number of topics per session’*.

(i) *‘Size of Village’:* The total population of Rusizi District is 32,313, with an average of 646 people per village. In the 50 intervention villages the population was 6942, with an average of 137 households per village. It was an oversight that the required size of village was not specifically spelt out in the protocol, i.e. that villages destined to be in the ‘Classic’ arm should have at least 100 households for the CHC facilitator to be able to achieve the specified target of > 70 members per CHC. As a result, village size varied, with only 5 CHCs achieving 100% coverage of all households. In fact 13 villages (26%) had considerably fewer than 100 households so could not meet the required number of members, whilst 17 villages were too large (over 150 households) for the CHC facilitator to achieve 80% coverage in the time allocated – an external determinant which prevented CHC facilitators from reaching targets (Table 6).
(ii) *Duration of Training Period*: Whilst the protocol specified a duration of 24 weeks (6 consecutive months) for the training component as per the manual, only 9 CHCs (18%) completed as per protocol as many sessions would have been cancelled or rushed due to daily torrential downpours. Table 6 shows that 20 CHCs (41%) had a duration of 17–19 weeks. The mistiming of the training to take place during the heavy rains was the singular most important effect of the research. Those 19 CHCs (39%) which had only 9–16 weeks were those who lost their facilitator due to local elections – an external determinant due to the political situation, which is again outside the control of local stakeholders.
(iii) *Number of Meetings:* The required target of 20+ meeting (sessions) was also compromised by the poor timing. There was a clear pattern of incomplete training: only 4 CHC (8%) met the minimal > 20 times (5 months), 8 CHCs (16%) met 17–19 times, whilst 29 CHCs (58%) met only 13–16 times, 9 CHCs (18%) met 9–12 times. None of the CHCs met less than 8 times (Table 6). A mean of 15 sessions were provided to the villagers, instead of 24 sessions as per protocol – a failure to meet specifications due to shortage of time, cannot be attributed to lack of community effort, as all sessions were provided were adequately attended, by on average over 41% of the membership.
(iv) *Number of Topics per Session: ‘Topic*’ refers to the content of each session as shown on the membership card (Table 2). According to the CBEHPP manual [7] each topic is designed to take 2 h so providing enough time for a participatory activity and dialogue in order to achieve maximum understanding of the issues discussed by the largely semi-literature membership. Although every CHC (100%) claimed to have provided all 24 topics, the cRCT report confused ‘topics’ with ‘sessions’. The detailed MoH records confirm that only 5 CHCs (10%) delivered one topic per session as directed. In fact, 16 CHCs (32%) doubled-up topics in 1–4 sessions, 23 CHCs (46%) doubled-up topics in 5–8 sessions in this manner, and 6 (12%) doubled-up in most sessions (9–12). This cramming of the syllabus can be attributed to an external determinant - lack of time. However, the fact all CHCs tried to complete all the topics at least indicates community interest despite an externally curtailed time frame. If the membership cards had been checked by the evaluation team it would have been clear from the date, that more than one topic was being done per session. Instead, to calculate attendance the evaluation team relied on recall from respondents (not necessarily CHC members) 1 year after the training. This gave an estimated mean of 9.5 meetings attended [10] with no clarity as to whether this referred to sessions or topics. MoH records show that whilst 18% of CHC provided 9 sessions, 58% of CHCs provided between 13 and 16 sessions.

**Table 6:** Indicators for Community Response levels in 50 villages in Rusizi District (2014–2015)

		# CHCs	% CHCS
Indicator 1:	SIZE: 100 households per village in a CHC		
High	> 200	3	6%
Good	151–199	14	28%
Average	101–150	20	40%
Low	> 100	13	26%
Indicator 2	DURATION: 24 consecutive weeks duration		
High	20+	9	18%
Good	17–19	20	41%
Average	9 to 16	19	39%
Low	< 8	1	2%
Indicator 3	NUMBER: 24 meetings in each CHC		
High	20+	4	8%
Good	17–19	8	16%
Average	13 to 16	29	58%
Low	9 to 12	9	18%
Fail	< 8	0	0%
Indicator 4:	TOPICS: Only one topic done per session		
High	1 topic per session	5	10%
Average	1–4 sessions with > 1 topic	16	32%
Low	5–8 sessions with > 1 topic	23	46%
Poor	9–12 sessions with > 1 topic	6	12%
		50	100%

### Process evaluation: expected inputs

The following is an analysis of how closely the intervention matched the protocol as specified in the CBEHPP manual [7] with five key inputs known as the 5 T’s: *(i) Training materials, (ii) Trainers, (iii) Training, (iv) Transport and (v) Timing.*

(i) *Training Materials’* consisted of a CBEHPP ‘Training Manual’ in Kinyarwanda for each CHC facilitator and a toolkit of over 300 visual aids for participatory dialogue sessions. As this material had previously been developed for national roll-out of the CBEHPP [6] and all *‘Training Materials’* were supplied on time as per protocol, we awarded the full 12/12 for this ‘input’.
(ii) *Trainers:* By the time the cRCT finally started in 2014, decisions at national level in the MoH had begun to negatively affect the CBEHPP. For example, MoH transferred responsibility for facilitating CHCs from their Village Health Workers (who are well versed in hygiene issues) to voluntary ‘Social Mobilisers’ (Affaires Sociales called ASOCs) under Local Government who are elected by the villagers. ASOCs have little, if any health background. Furthermore, after having just been trained to become CHC facilitators for the intervention in Rusizi, one third of the 50 ASOCs were not re-elected, thus further delaying the start of the intervention. EHOs had to visit the ASOCs and train them in situ, which reduced the quality and quantity of training provided. Due to the reduced quality of the type of trainers and there was a need for more supervision than had been planned to assist new and inexperienced ASOCs. Therefore only 10/24 awards were awarded for *‘Trainers’*.
(iii) *Training:* For CHCs to achieve ‘buy-in’ for recommended changes, it is considered critical that a dialogue is generated to ensure that problem-solving is done by the participants and solutions are not handed out as a directive from the teacher. This is achieved by a series of carefully developed participatory activities, using a ‘tool kit’ of visual aids to provoke discussion, which allows even illiterate people to join in without fear. Each session is designed to take 2 hours to ensure enough time for debate and group resolution. Although all topics were done, the facilitators crammed more than one topic into each session in order to complete the course. With tropical downpours falling most afternoons, we surmise topics would have to be rushed, with resort to a didactic style of delivery although less time-consuming is also less likely to generate dialogue and consequent group commitment. Lack of experience in hygiene issues by ASOCs also reduced the quality of the community training, therefore 17/32 was awarded.
(iv) *Transport:* EHOs are normally based in District Health Centres or the district hospital. They had no dedicated transport which was essential to monitor the remote project villages that had been randomly selected from across the entire area of Rusizi District (960 sq. kms with 596 villages). Due to bureaucratic delays, the motorcycles were finally provided only *after* the intervention. The budget provided directly to the MoH to cover EHO transport costs did not reach the district, thereby grounding district staff who had no fuel allowance for motorcycles. These constraints would be less likely to have derailed an intervention if the implementation had been done by a well-resourced international NGO, (such as WaterAid) [19] with proper support and staff capacity as has been shown by other studies of CHCs in Rwanda over the same period [18, 20, 21]. As EHOs had neither motorcycles nor fuel during the critical 5-month period of the intervention, mobility was clearly lacking so 6/12 is a generous score for *‘Transport’*. Although bicycles were provided to ASOCs, these proved to be inappropriate for use as transport due to the mountainous and wet terrain.
(v) *Timing:* The season when the training took place (February – July) could not have been a worse time for community training, being the height of the long rains (over 1300 mm per year). Despite the late start of the intervention, due to cRCT delay in completion of base line, which cut short implementation time by six months the research team still expected the implementation team to complete the CHC training of the communities on time. Thus Year 1 of the intervention was reduced from 12 months to 5 months. During the dry season (June–December) large groups of 50–100 people can attend CHC training by sitting under a shady tree, but in the rains as there are few large meeting halls so they must seek shelter in small huts. With CHC members sometimes walking up to a kilometre to get to the venue on steep, slippery paths, attendance is likely to have been affected. A generous score of 9/20 was allocated for ‘*Timing’*.

### External Determinants

The explanations by Environmental Health Officers and other district leaders in the Focus Group Discussions as to the reasons for some failures of the intervention were grouped into two principal categories of external determinants: (a) the effect of Government and (b) the effect of the cRCT research itself.

(i) *The Effect of Government on the Intervention*

This case study of CBEHPP as a national programme over the past 8 years, demonstrates clearly that unless a programme is well supported by leaders in Ministry of Health at national level and at a district level by Local Government, then there is little chance of it being successful at village level [11]. During the intervention year, the Environmental Health Desk at national level was temporarily moved to the Bio-Medical Centre (2014–2015) leaving a leadership vacuum with no committed CBEHPP ‘champion’ at national level exactly during the time when the intervention was being evaluated, and support for Local Authority was correspondingly low in Rusizi District. CBEHPP nationally was able to recover its previous momentum again in 2016 (which was after the cRCT end-line in Rusizi had been completed) and CHCs in Rusizi again resurged. In 2017, eight new districts were funded by USAID & UNICEF for an Integrated Nutrition-WASH programme (INWA) using existing CHCs to reduce stunting. Despite the operational challenges imposed during the evaluation period (2013–2015), it was found by an internal government assessment that, Rusizi District had risen in the ranking from being 4th from the *bottom* in the country, to 4th from the *top* of 30 districts in terms of a healthy environment and received a national award at the annual *Imihigo* performance assessment [22], being cited as one of the Districts with a high score (98.5%) for ‘Monitoring and Mobilization’ of communities. Government monitoring and the effect it has on the success of a national programme is one of the main drivers of development, and the Rwanda system of *Imihigo* appears to be a successful strategy to ensure high performance from the Local Authority. Based on the results of local monitoring, the Rwandan government has since expanded both the scope and the reach of the CBEHPP throughout Rwanda.

(ii) Effects of the Research on the intervention

The cRCT design specifically aimed to ensure no CHC village shared a common border with another village to reduce the risk of ‘contamination’. However, the artificial spacing of villages in this manner is not how a ‘normal’ CHC project would be designed, as rapid diffusion of innovation to reach a critical mass is achieved more effectively by clustering of villages shown to be the most effective way to contain transmission of diseases [23]. Isolating villages across a mountainous district of 960km^2^ also posed major logistical challenges for monitoring CHCs given the shortage of transport and is likely to have affected the uptake. The most critical effect of the cRCT research on the intervention was the timing of the training in the villages. The urgency to complete the end-line survey was dictated by the need to maintain those children enrolled in the cohort before they grew too old to be remeasured. This put undue pressure on the intervention to adhere to the research time frame which resulted in a loss of quality and significant curtailment of the training period and vital follow-on graduations and CHC household competitions. Normally, after the end of the CHC training period, there are at least 6 months of follow-up by field staff to organise inter-club competitions and graduation ceremonies for each CHC [4, 5, 7–9]. Instead, this vital stage was skipped when the research team insisted all AA field staff should withdraw from the district as per the original timing of the schedule, despite having been responsible for the six-month delay in start-up. Monitoring which should have been done by the EHOs was not done adequately as they were largely grounded due to lack of transport. As a result, there were fewer ‘graduations’ and no ‘model home’ competitions were held. With such little visibility of CHC activity in the village to inspire new members to join, membership did not spread as well as planned.' Inflexibility and the lack of accommodation to enable this social trigger by the cRCT, again undermined the effect of positive peer pressure to trigger high levels of social mobilisation [9]. Inflexibility and the lack of accommodation to enable this social trigger by the cRCT, undermined the mechanism of positive peer pressure, which is one of the principal mechanisms of social change of the CHC model [4, 13].

## Discussion

When evaluating the impact on health of the Community Health Model in Rusizi District, it is important to bear in mind that the *spread* of training within Classic villages was on average only 58% with only 5 CHCs (10%) in full compliance to training standards for a Classic CHC. Of those registered CHC households consisting of 4016 CHC members in total, less than half (42%) had completed the full 20 session training by the time the cRCT end-line was taken. It would therefore appear that the end line was taken prematurely, as completion of training is a basic requirement if any reduction of diarrhoea in a household can be expected using the CHC Model [23]. This is because the blocking of the transmission of diarrhoea requires a raft of hygiene changes which are implemented in incremental stages throughout the home and yard of the CHC member. For this to happen, the CHC member must change her whole attitude to her standard of hygiene, involving a lot more effort than normal to reach such hygiene standards. There are no short cuts to developing such a Culture of Health which is a shift in the basic understanding and priorities of the entire community [9]. Time is needed for positive reinforcement of good hygiene standards by the critical mass of the village which may take well over a year to achieve [8] and in the case of Rusizi it took 3 years [18]. The shortage of time to complete the training was keenly observed at the Focus Group Discussion with practitioners insisting that pressure due to research timing had negatively affected the delivery, such that the intervention deviated significantly from what was had been planned prior to intervention.

What is more difficult to understand is how the *measurement* of the impact on behaviour change could have gone awry in the per-protocol assessment. From the perspective of the implementing team, the reason may be that the main independent variable (i.e. number of health sessions attended) was miscalculated. When conducting a survey in a CHC household, it would have been critical to ensure that the *exact* number of sessions attended were correctly calculated and to know *which* topics of the health promotion sessions were covered by the household. In the cRCT end line survey it was unreliable to use the *recall* of the respondent who may or may not have been the CHC member who attended the sessions. For example, if the respondent was a male head of household, it was likely that his wife was the person who attended the CHC sessions because over 80% of CHC members were women. Therefore, to prompt him to identify which sessions had been attended by his wife 1 year after the training, was unlikely to yield accurate information.

Although it was claimed that the CHC Membership cards were difficult to locate in each home, this was found to be untrue when the Membership Cards were collected from virtually all CHC households by the monitoring team within a month from all the 50 classic CHCs in Rusizi District. That these Membership Cards, upon which the CHC model is based, were not used by cRCT to triangulate against records held by all the CHC facilitators seems to have been a critical oversight which is inadequately justified given this system of community monitoring is intrinsic to the design of the CHC Model [8]. Another objection to the cRCT findings by those implementing the intervention was that the cRCT survey tool was not fully compatible with the Ministry of Health monitoring tool for CBEHPP with indicators specific to the Rwandan context [7]. These indicators are clearly laid out in the CBEHPP Training Manual and were also reflected in the CHC Membership cards (Table 2). For the past 20 years all CHC programmes started by Africa AHEAD, had used standard proxy indicators to evaluate the impact of the training on CHC members which is well documented in the literature [5, 9, 8, 23].

Apart from the seven standard WASH indicators discussed above, which were not adequately adapted to the Rwandan context, there was a heavy emphasis in the cRCT findings on nutritional status of children i.e. the reduction of stunting as an indicator of the health impact of the intervention [10]. The inclusion of nutritional indicators was in fact, inappropriate given that improved nutrition of children is not one of the expected health outcomes in the first stage of a CHC. This is because in the first year of training the main objective is to achieve general hygiene in the home and safe sanitation, whilst there are no practical inputs to ensure a balanced diet with increased food production. In the full life of a CHC as described in the AHEAD Model [8] nutrition is addressed comprehensibly in the third year when CHCs may convert into FAN (Food Agriculture and Nutrition) Clubs [8]. To expect an effect on stunting in children under 5, after only two short nutrition theory sessions was unrealistic, especially when measuring the impact 1 year after the end of the training. That the evaluation expended considerable time to assess the stunting levels of almost 8000 children, measuring length-for-age Z score and weight-for-length Z score was an extravagant use of resources, which also critically delayed the start-up of the intervention so undermining the quality of the training. Stunting in babies under 1 year of age (i.e. those born after the CHC training was completed) was 23.5% in the Classic CHC villages compared to 23% the Lite and 27% in the Control - a 3.5% improvement which could have been interpreted as a positive trend by the cRCT, given such minimal input on nutrition. However, the cRCT also found that there was no difference between three arms in stunting in children under 2 (those born during the training period) which was 37%; whilst stunting in children under 5 (who were already 3 years old when the CHC training was done) was difficult to explain being lower at 41% in both in Classic and in the Control than in the Lite which showed 43%. Apart from showing how stunting increases as the toddler grows there is no logical pattern to explain the difference between the arms, and more contextual analysis is needed to understand such counter-intuitive findings. Leading authorities on the methodology of evaluation [11] have identified how many of these failures in the field could have been avoided, by including the use of monitoring data as a reliable means of triangulation but there was almost no communication between the two teams. Given the scarcity of resources in the WASH sector to achieve safe hygiene and sanitation for all, better support for achieving more accurate monitoring data within Ministry of Health which stimulates higher community response in the long-term, would be more cost-effective, than investment in extensive external evaluations.

In the last few years, three similar cRCT’s measuring the impact of standard WASH interventions in Zimbabwe, Kenya and Bangladesh, also apparently failed to find much impact on child health despite high implementation fidelity [2]. Whilst academics maintain the ‘biological plausibility of WASH as public health interventions is not challenged by these findings’ and reiterate the well established fact that ‘ingestion of human faeces is hazardous to human health’, the United Nations Sustainable Development Goal targets, are requiring more long-term holistic ‘WASH++’ interventions to reduce diarrhoea and stunting, whilst others highlight how long it has taken well developed countries to reach the required standard of hygiene to control such disease [14, 24]. Based on the findings in this paper, we once more emphasize that Community Health Clubs are an ideal vehicle for such ‘transformative WASH’ interventions as the model is more holistic than other mobilization strategies used purely to achieve targets in the WASH Sector such as PHAST [16] and CLTS [17]. Sustainability is critical when evaluating cost-effectiveness, as reversion to previous high-risk practices negate the gains of a program [25]. On closer examination using monitoring data over a longer period, the CHC intervention in Rusizi was much more resilient than the impressions given by the cRCT findings [10]. The fact that all 50 CHCs continued training members unsupervised more than 30 months after the end of the intervention, supplying an extra 379 revision sessions at no extra cost to the donors, demonstrates a note-worthy sense of local ownership; it also indicates a community mobilisation model that is self-reliant. In spite of poor timing of the intervention, despite minimal transport for monitoring and a curtailed intervention period and with no external budget for home improvements, over 70% of CHC members made healthy choices towards improving their living conditions achieving all seven indicators used by IPA in the cRCT [10] within roughly 3 years, as shown in this paper. Such lack of donor dependence would be generally understood by experienced practitioners to reflect a high level of ‘sustainability’ of the CHC as a village structure [25]. Our recent comparative study between CHCs in Zimbabwe and the cRCT Intervention in Rwanda also shows that whilst there is still a gap in hygiene standards between the interventions in the two countries, the level of response from the Rusizi community is not far behind that of Zimbabwe, where the methodology was started 25 years ago [18]. Other studies from Rwanda [20], Democratic Republic of Congo [26] and Bugesera District in Rwanda [19] find similar levels of response as those identified by our monitoring data in Rusizi District. Leading NGOs in Rwanda are not only scaling-up but also extending the scope of CHC activity to meet the demands of the Sustainable Development Goals (UN, 2016) in gender, employment, nutrition, water and sanitation and as a by product generating increased social capital and women’s empowerment (24). Community Health Clubs are also being used by other NGOs within the region, including in the most fragile of communities in DRC [26] providing more experience in emergency use of the CHC Model in post conflict areas, as well as for emergency relief and cholera mitigation in urban areas of Haiti [27].

The Public Health sector would benefit from more cross-country analysis of the different CHC interventions, comparing monitoring data to understand the impact of local context [18], as well as measuring the cost effectiveness of the CHC Model with other different mobilisation strategies such as Community Led Total Sanitation [25]. The CHC methodology should not be summarily dismissed as ineffectual as a result of a single cRCT, particularly at a time when the WASH sector is searching for a way to provide more comprehensive additions to broaden standard water, hygiene and sanitation programs.

## Conclusions

Monitoring data provides coherent explanations for the perceived lack of health impact in the Rusizi intervention, mainly related to external determinants. Such community-based assessment is also able to accurately measure community response and shows that Community Health Clubs had considerable resilience despite numerous challenges which were not adequately considered in the external evaluation which missed much of the context. The cRCT conclusion that the case study of Rusizi District does not encourage the use of the CHC model for scaling up, raises concerns over the possible misrepresentation of the potential of the holistic CHC model to achieve health impact in a more realistic time frame. It also raises questions as to the appropriateness of apparently rigorous quantitative research, such as the cluster-Randomised Controlled Trial as conducted in Rusizi District, to adequately assess community dynamics in complex interventions.

## Supplementary information

**Additional file 1.** Househole Inventory Monitoring Tool.

**Additional file 2.** Indicators of Community Response.

## Data Availability

All data is available upon request to the corresponding author.

## Abbreviations

(Africa) AHEAD: Applied Health Education and Development
ASOC: Affaires Sociales
CBEHPP: Community Based Environmental Health Promotion Programme
CHC: Community Health Clubs
CLTS: Community Led Total Sanitation
cRCT: Cluster Randomised Controlled Trial
DRC: Democratic Republic of Congo
EH: Environmental Health
EHO: Environmental Health Officer
HSSP: Health Sector Strategic Plan
IPA: Innovations for Poverty Action
MDG: Millennium Development Goal
MoH: Ministry of Health
NGO: Non-Government Organisation
ODK: Open Data Kit
PHAST: Participatory Hygiene and Sanitation Transformation
WASH: Water, Sanitation and Hygiene
ZOD: Zero Open Defecation
