# Supplementary material for: The value of monitoring data in a process evaluation of hygiene behaviour change in Community Health Clubs to explain findings from a cluster-randomised controlled trial in Rwanda
Source: BMC Public Health. 2020 Jan 23;20:98. doi: 10.1186/s12889-019-7991-7 (PMC6979057; doi:10.1186/s12889-019-7991-7)
Supplement: Supplementary file 1 — Additional file 1. Househole Inventory Monitoring Tool. [file 12889_2019_7991_MOESM1_ESM.pdf]

HOUSEHOLD INVENTORY MONITORING TOOL

Developed by Africa AHEAD for the Ministry of Health Rwanda (2013 - 2017)

Community Based Environmental Health Promotion Programme (CBEHPP)

Technical Assistance of Mobile Technology - Overtone

**Mobile Data Collection Tools v2.0 - Content Design Specifications**			

Q Code	*Title*	*Explanation*	A #	*Multiple choice*	Required	Traffic Light
MID	Device ID				*	
MStart	Start Time				*	
MEnd	End Time				*	
E1	Name of enumerator	*Please write your family name and first name .*			*	
E2	Enumerator’s code	*Please write your code.*			*	
LN1	Country name				*	
LN2	District name				*	
LN3	Sector / Ward name				*	
LN4	Cell name				*	
LN5	Village name				*	
LN6	CHC name					
LN7	Confirm you have entered the correct location.	Swipe to next question if this is correct or go back to edit.				
LC1	Country code				*	
LC2	District code				*	
LC3	Sector / Ward code				*	

LC4	Cell code				*	
LC5	Village code				*	
LC6	CHC code	*If not a CHC write "0".*			*	
LC7	Confirm you have entered the correct CHC code.	Swipe to next question if this full code is correct or go back to edit.			*	
ST1	Type of survey	*Please select one of the below.*			*	
			Base	Base line		
			Mid	Mid line		
			End	End line		
			Monthly	Monthly		
			Annual	Annually		
			Test	Test		
ST2	Training level	*Please select which type of training this village has received.*			*	
			Classic	Classic		
			Lite	Lite		
			None	None		
ST3	Settlement type	*Note where the household is located.*			*	
			Urban	Urban		
			Peri Urban	Peri Urban		
			Rural	Rural		
			Camp	Camp (IDP / Refugee)		
ST4	Are any of these services provided to this area?	*If applicable select more than one. If not sure or don't know then skip this question.*				
			Water	Water		
			Electricity	Electricity		
			Garbage	Garbage collection		
			None	No services provided		
ST5	What is the household's GPS location?	*Ensure location / GPS positioning is enabled on the device to set current location.*				

PA1	*Observe access to main household.*				*	
			Flat	Flat land		
			Steep	Steep land		
PA2	*Observe if access is safe when path is wet.*				*	
			Safe	Safe		
			Not Safe	Not safe		
CD1	Is your household registered in a Community Health Club?				*	
			Registered	Yes, registered		
			Not Registered	No, not registered		
CD2	If yes, is your CHC still active?				*	
			Active	Yes		
			Not Active	No		
CD3	When was the last meeting?				*	
			Week	Within the last week		
			2 Weeks	Within the last two weeks		
			Month	Within the last month		
			3 Months	Within the last 3 months		
			6 months	More than 6 months		
			Year	More than a year		
			2 Years	More than 2 years		
			NA	Cannot remember / Do not know		
CD4	May I see your membership card and certificate (if applicable)?	*Observe how many sessions have been completed at time of data collection. Enter number.*			*	
DH0	What is the name of the head of the household?	Please write full first name and surname.				
DH1	What is the religion of the head of the household?				*	

			Christian	Christian		
			Moslem	Moslem		
			Other	Other		
DH2	Total males in household.	*All males who regularly eat from the same pot. Enter number.*			*	
DH3	Total females in household.	*All females who regularly eat from the same pot. Enter number.*			*	
DH4	Total number of people in the household?	*All household members (male and female) who regularly eat together from the same pot. Confirm that this is the total number of people.*				
DH5	What gender is the respondent?	*If possible interview the 'main woman'. If there is no 'main woman' then interview the male head of household.*			*	
			Female	Female		
			Male	Male		
DH6	What is the approximate age of the respondent in years?	*Enter "0" if not known. Leave empty if not willing to answer.*				
DH7	What is the level of education of the respondent?				*	
			Some Primary	Some primary school		
			Primary	Completed primary school		
			Some Secondary	Some secondary school		
			Secondary	Completed secondary school		
			Further Education	Further education		
			Training	Other training		
			None	No education		
			NA	Not applicable / not willing to answer		
ST6	Does this household have children under 18 years old?	*You will be asked to enter the number of children in the following questions. Enter 'No' if not applicable.*			*	
			Yes	Yes		
			No	No		
C1	How many children are of 2 years or under?	*If not applicable or no children in this age group enter "0".*			*	
C2	MUAC	*Measure the Upper Arm Circumference (MUAC) of one child between 0 - 2 years old.*				
			Green	Green		
			Yellow	Yellow		

			Red	Red		
			NA	Not possible		
C3	How many children between the ages of 3 – 5 years old?	*If not applicable or no children in this age group enter "0".*			*	
C4	MUAC	*Measure the Upper Arm Circumference (MUAC) of one child between 3 - 5 years old.*				
			Green	Green		
			Yellow	Yellow		
			Red	Red		
			NA	Not possible		
C5	What is the total number of children under 5 years old?	*Check that the number of children under 5 is correct. If not, go back and correct it.*				
C6	What is the total number of children under 5 years old that are properly immunised?	*Ask to see the Road to Health Card and check to see if all children are immunised. If no card write '0" .*			*	
C7	How many children are between 6 - 10 years old?	*If not applicable or no children in this age group enter "0".*			*	
C8	How many children are between 11 - 18 years old?	*If not applicable or no children in this age group enter "0".*			*	
C9	Total number of childen under 18 years old	*Check that the number of children under 18 is correct. If not, go back and correct it.*				
C10	How many of these children are orphans?	*If not applicable enter "0".*			*	
G1H1	1 HOUSE TYPE	*Observe the building materials used to construct the house / hut.*			*	
			1	Permanent house		
			2	Burnt brick house		
			4	Semi permanent house / Mud bricks/ hut		
			8	Mud / pole hut / house		
			16	Shack		
G1H2	2 ROOF	*Observe the roofing materials.*			*	
			1	Permanent (Iron sheets / tiles) and ceiling / no holes		
			2	Permanent (Iron sheets / tiles) no ceiling / holes in roof		
			4	Temporary (thatch) / some parts are iron / tiles		

			8	Temporary (thatch only) good condition / no holes		
			16	Temporary (thatch only) needs re-thatching / holes		
G1H3	3 FLOOR	*Ask the respondent if you can see inside the house and observe the floor.*			*	
			1	Cement / concrete		
			2	Mud / cement patchy		
			4	Mud with cement smeared		
			8	Mud with cow dung smeared		
			16	Only mud		
G1H4	4 WALLS	*Observe the walls.*			*	
			1	Plastered with cement / painted		
			2	Brick plastered		
			4	Brick unplastered		
			8	Mud plastered		
			16	Tin / planks		
**G1HX**	Has the household scored RED in any of the Housing questions?	If yes, then this household is at risk and needs improvement.	YES or NO			
**G1HY**	Has the household scored YELLOW in any of the Housing questions?	If yes, then this household has made progress but needs further improvement.	YES or NO			
**G1HZ**	HOUSING - LEVEL ACHIEVED20/10/2019: RED, YELLOW or GREEN	The household has achieved the following score for this indicator.<br>If Red, household is at risk and needs to improve.<br>If Yellow, household has made progress but needs further improvement.<br>If Green, congratulate household as they have achieved excellent hygiene standards for this indicator.				
G2C0	COMPOUND CASES	*Observe if household has its own compound.*<br>*If yes, ask to be shown around and observe:*<br>*Latrine, hand washing facility, shower / washroom, kitchen, cooking area, pot rack, livestock stalls, drainage, vegetables, fruit trees, any open defecation, animal dung.*			*	
			Yes	Yes		
			Shared	Shared		
			No	No		

G2C1	1 WATER DRAINAGE	*Observe how rainwater / grey (soapy) water and black water (livestock sludge) is drained around the house.*			*	
			1	Extensive drainage channelled into covered pit / tank / soakaway		
			2	Extensive drainage channelled into open pit / plantation		
			4	Some drainage but not effective / standing grey water		
			8	Drainage into unsafe pit / standing black water		
			16	No drainage / some erosion seen / standing rainwater in pot holes		
G2C2	2 GREENING	*Observe the effort of beautification of the compound. If applicable select more than one.*			*	
			1	Flowers planted / pot plants		
			2	Shade trees / shrubs and hedges		
			4	Compound covered with grass		
			8	Grass in patches		
			16	No shade trees / shrubs and hedges		
			32	No effort		
G2C3	3 SOLID WASTE: SWEEPING	*Observe if the area around the house is well swept.*			*	
			1	Completely swept		
			2	Well swept		
			4	Swept in some places		
			8	Very little sweeping		
			16	No sweeping		
G2C4	4 SOLID WASTE: TYPE	*Observe what type of waste is thrown around the house.*			*	
			1	No waste of any kind seen in yard		
			2	Very little waste of any kind seen in yard		
			4	Some rotten vegetable waste seen, a few papers		
			8	Rotten vegetable waste, plus some paper, plastic, bottles / tins lying around		
			16	A lot of rotten vegetable waste, and a lot of paper, plastic, tins & bottles		

G2C5	5 SOLID WASTE MANAGEMENT	*Observe how solid waste is disposed.*			*	
			1	Municipal garbage collection service		
			2	Solid waste separated / recycled in different ways		
			4	Solid waste disposed in dedicated area: pit / fenced / roofed area		
			8	Some attempt at disposal in dedicated area and managed / burnt		
			16	Some attempt at disposal in dedicated area but NOT managed / burnt		
			32	No attempt at solid waste disposal		
**G2CX**	Has the household scored RED in any of the Compound questions?	If yes, then this household is at risk and needs improvement.	YES or NO			
**G2CY**	Has the household scored YELLOW in any of the Compound questions?	If yes, then this household has made progress but needs further improvement.	YES or NO			
**G2CZ**	COMPOUND - LEVEL ACHIEVED: RED, YELLOW or GREEN	The household has achieved the following score for this indicator.<br>If Red, household is at risk and needs to improve.<br>If Yellow, household has made progress but needs further improvement.<br>If Green, congratulate household as they have achieved excellent hygiene standards for this indicator.G195				
**G3W0**	**WATER SOURCE CASES**	*Is this household connected to the piped water supply by the Municipality or Council? Observe and ask.*			*	
			Yes	Yes		
			No	No		
G3W1	**1 TYPE - What is your primary water source?**	*Observe and ask.*			*	
			1	Safe piped water / tap / tank		
			2	Water kiosk		
			4	Borehole / hand pump		
			8	Protected spring		
			16	Protected well		
			32	Protected tank		
			64	Protected rainwater tank		

			128	Contaminated borehole		
			256	Unprotected open well		
			512	Unprotected spring		
			1024	Open rainwater tank		
			2048	River, stream, dam, rain puddles		
G3W2	**2 RELIABILITY - Is your selected primary source reliable all year round?**	Ask.			*	
			1	Water supply all year		
			2	Slow recharge occasional shortage / low water table		
			4	Shortage in dry season, no safe alternative		
			8	Water supply cut off / out of order, no safe alternative		
G3W3	**3 DISTANCE TO WATER SOURCE - How long does it take to walk to the water source one way?**	Ask.			*	
			1	None / water source in compound		
			2	Less than quarter of an hour		
			4	Half an hour or less		
			8	More than half an hour		
			16	More than an hour		
G3W4	**4 WAITING TIME AT WATER SOURCE - How long does it take to queue for water?**	Ask.			*	
			1	No waiting time		
			2	Less than quarter of an hour		
			4	Half an hour or less		
			8	More than half an hour		
			16	More than an hour		
G3W5	**5 AMOUNT COLLECTED - How many liters of water are collected by the household daily?**	For example: 1 container = 20 liters			*	
G3W6	Total litres per household member per day.	*This calculation shows the amount of water per person in the household each day.*			*	

G3W7	Enough water per household member each day?	*Green = More than 15 liters. Yellow = 15 liters. Red = less than 15 liters.*			*	
			GREEN			
			YELLOW			
			RED			
G3WX	Has the household scored RED in any of the Water Source questions?	If yes, then this household is at risk and needs improvement.	YES or NO			
G3WY	Has the household scored YELLOW in any of the Water Source questions?	If yes, then this household has made progress but needs further improvement.	YES or NO			
G3WZ	WATER SOURCE - LEVEL ACHIEVED: RED, YELLOW or GREEN	The household has achieved the following score for this indicator.<br>If Red, household is at risk and needs to improve.<br>If Yellow, household has made progress but needs further improvement.<br>If Green, congratulate household as they have achieved excellent hygiene standards for this indicator.				
G4D0	DRINKING WATER STORAGE CASES - Do you store your drinking water?	*Observe the drinking water.*			*	
			Yes	Yes		
			No	No		
G4D1	**1 DRINKING WATER STORAGE**	*Observe how the drinking water is stored.*			*	
			1	Stored in water filter		
			2	Bottled drinking water purchased		
			4	Stored in covered bucket / closed jerry can		
			8	Stored in poorly covered bucket / clay pot / other		
			16	Stored in open / uncovered bucket / clay pot / other		
G4D2	**2 HYGIENE**	*Observe cleanliness of drinking water container.*			*	
			1	Very clean inside and outside		
			2	Quite clean inside and outside		
			4	Quite clean inside and dirty outside		

			8	Dirty and algae inside, but stored inside the house		
			16	Very dirty inside and left outside		
G4D3	**3 TREATMENT - Do you treat your drinking water?**	Observe and ask.			*	
			1	Well boiled		
			2	Solar		
			4	Chemically Sur L'eau /Jik and / or filter		
			8	Some of the above		
			16	Not treated as water is said to be from safe source		
			32	None of the above / although water is unsafe		
G4D4	**4 USAGE** - Please can you give me a drink of water?	*Observe how drinking water is served.*			*	
			1	Poured straight from filter / container / tap		
			2	Taken with a jug / ladle		
			4	Take with a cup and put into another cup to drink		
			8	Taken with a cup and consumed with the same cup		
			16	Taken and consumed directly by hand / drink from container		
**G4D5**	**5 OBSERVE POINT OF USE WATER CLARITY**	*Observe the water clarity.*			*	
			1	Very safe: very clear		
			2	Safe: looks quite clear		
			4	Dangerous: turbid, muddy		
**G4DX**	Has the household scored RED in any of the Drinking Water Storage questions?	If yes, then this household is at risk and needs improvement.	YES or NO			
**G4DY**	Has the household scored YELLOW in any of the Drinking Water Storage questions?	If yes, then this household has made progress but needs further improvement.	YES or NO			
**G4DZ**	**DRINKING WATER STORAGE - LEVEL ACHIEVED: RED, YELLOW or GREEN**	The household has achieved the following score for this indicator.<br>If Red, household is at risk and needs to improve.<br>If Yellow, household has made progress but needs further improvement.<br>If Green, congratulate household as they have achieved excellent hygiene standards for this indicator.				

G5HW1	1 HAND WASHING - METHOD	*If any children present, ask them to wash their hands. If not, ask respondent.*			*	
			1	Excellent: rub each finger for long time (about 30 seconds) and nails, soap used		
			2	Good: hands rubbed well, soap used		
			4	Average: quick wash, soap used		
			8	Poor: reuse of water in a bowl, no soap used		
			16	Very bad: shared bowl of used water, no soap		
			32	No hand washing practised		
G5HW2	2 PLACE	*Observe where hand washing facilities are.*			*	
			1	Hand washing facility by kitchen and toilet or more		
			2	Hand washing facility by toilet		
			4	Hand washing facility in home (basin / jug)		
			8	No fixed place for hand washing facility		
			16	No hand wash facilities at all		
G5HW3	3 HAND WASH FACILITY DESIGN	*Observe the type of hand washing facility.*			*	
			1	Tap		
			2	Step & Wash / Canacla		
			4	Permanent (manufactured)		
			8	Temporary (homemade Tippy tap)		
			16	Pour to waste over basin		
			32	Jerry can		
			64	Plastic bottle		
			128	Common bowl		
G5HW4	4 USAGE OF HAND WASHING FACILITY	*Observe if they are used.*			*	
			1	In use / filled with water		
			2	Evidence of use but not with water		
			4	Broken / no water		

G5HW5	5 AVAILABILITY OF SOAP / ASH	*Observe if there is soap present.*			*	
			1	Soap at hand washing facility and used		
			2	Ash at hand washing facility and used		
			4	Soap / ash available in house, but not at hand washing facility		
			8	No soap seen, but say they usually use soap		
			16	No soap available / ash not used		
**G5HWX**	Has the household scored RED in any of the Hand Washing questions?	If yes, then this household is at risk and needs improvement.	YES or NO			
**G5HWY**	Has the household scored YELLOW in any of the Hand Washing questions?	If yes, then this household has made progress but needs further improvement.	YES or NO			
**G5HWZ**	RED, YELLOW or GREEN	*The household has achieved the following score for this indicator.*<br>*If Red, household is at risk and needs to improve.*<br>*If Yellow, household has made progress but needs further improvement.*<br>*If Green, congratulate household as they have achieved excellent hygiene standards for this indicator.*				
**G6S0**	**SANITATION CASES - Do you have access to a latrine (dug hole in the ground) or a toilet (manufactured facility)?**	*Observe and ask.*			*	
			Yes	Yes		
			No	No		
G6S1	**1 LATRINE OWNERSHIP - Is the latrine used only by your own family?**	*Observe and ask.*			*	
			1	Own latrine / toilet		
			2	Communal ablution		
			4	Shared latrine / toilet with neighbour		
G6S2	2 LATRINE TYPE	*Determine the type of latrine / toilet.*			*	
			1	Water closet / WC (Flush toilet connected to sewer/soak away)		
			2	Pour Flush Toilet (water used to seal toilet)		
			4	EcoSan latrine (urine separation or diversion / dry)		

			8	Ventilated Improved Pit latrine VIP (permanent)		
			16	Permanent covered pit latrine (PCP) (permanent slab / sanplat /cover/ walls / floor)		
			32	Ventilated Improved Pit Latrine (temporary, local materials)		
			64	Pit latrine with cement slab or sanplat (no cover)		
			128	Traditional pit with floor of logs / mud (with cover)		
			256	Traditional pit with floor of logs / mud (no cover)		
G6S3	3 NO LATRINE	*If no latrine present, observe what type of sanitation is practised.*			*	
			1	Cat sanitation		
			2	Recycling (biodegradable sealed bag)		
			4	Flying toilet (plastic bag)		
			8	Open defecation (adults / children)		
			16	Toddlers faeces left lying without disposal in latrine		
G6S4	4 PIT LATRINE FLY CONTROL	*Observe if flies are controlled properly.*			*	
			1	Use of ventilation pipe (working) on latrine pit		
			2	Pit latrine with well fitted cover		
			4	Latrine lid is quite well fitted but there are still flies		
			8	Use of ventilation pipe but broken / no gauze		
			16	Pit latrine with poorly fitted cover		
			32	Pit latrine with cover but not used		
G6S5	5 CLEANLINESS	*Observe the cleanliness of the latrine.*			*	
			1	Very Good: hygienic (no smell, urine, faeces nor flies seen)		
			2	Good: No faeces, urine nor flies seen		
			4	Average: No faeces nor urine, but smelly		
			8	Bad: Smelly, urine only / flies		
			16	Very bad: Smelly, very dirty, faeces / urine / flies		
**G6SX**	Has the household scored RED in any of the Sanitation questions?	If yes, then this household is at risk and needs improvement.	YES or NO			

**G6SY**	Has the household scored YELLOW in any of the Sanitation questions?	If yes, then this household has made progress but needs further improvement.	YES or NO			
**G6SZ**	RED, YELLOW or GREEN	*The household has achieved the following score for this indicator. If Red, household is at risk and needs to improve. If Yellow, household has made progress but needs further improvement. If Green, congratulate household as they have achieved excellent hygiene standards for this indicator.*				
G7B1	1 PLACE FOR BODY WASHING	*Observe the wash place.*			*	
			1	Private wash room in house		
			2	Permanent wash room outside		
			4	Temporary wash shelter outside		
			8	Wash place outside but no privacy		
			16	No evidence of washing at home		
G7B2	2 USE SOAP FOR BODY	*Observe if there is soap at the wash place.*			*	
			1	Soap at wash place		
			2	Soap available but not at wash place		
			4	No soap seen, but they say they use soap		
			8	No soap available		
G7B3	**3 CLOTHING CLEANLINESS**	*Observe the state of adults' clothes.*			*	
			1	Adults in clean clothes		
			2	Adults in fairly clean clothes		
			4	Adults in dirty clothes		
G7B4	**4 BEDROOM TIDYNESS**	*Observe if the bedroom is tidy, ie if clothes are stored in cupboard or container and not left on the floor.*			*	
			1	The bedrooms are tidy, clothes are well stored		
			2	The bedrooms are quite tidy, some clothes left around		
			4	The bedrooms are very disorderly with no clothes stored		
			8	Only mats for sleeping / clothes very badly stored		
G7B5	**5 BED FACILITIES**	*Observe the sleeping faclities.*			*	

			1	Beds with matresses		
			2	Mats on floor		
			4	Blankets on the floor		
**G7BX**	Has the household scored RED in any of the Body Hygiene questions?	If yes, then this household is at risk and needs improvement.	YES or NO			
**G7BY**	Has the household scored YELLOW in any of the Body Hygiene questions?	If yes, then this household has made progress but needs further improvement.	YES or NO			
**G7BZ**	RED, YELLOW or GREEN	*The household has achieved the following score for this indicator. If Red, household is at risk and needs to improve. If Yellow, household has made progress but needs further improvement. If Green, congratulate household as they have achieved excellent hygiene standards for this indicator.*				
**G8M0**	**MALARIA PROTECTION CASES - Has any member of your household had malaria in the last year?**	Ask.			*	
			No	No		
			Yes	Yes		
G8M1	**1 PROTECTION METHOD -** What do you use to prevent malaria?	*Select more than one method if applicable.*			*	
			1	Malaria prophylaxis		
			2	Mosquito bed nets		
			4	Netting on window		
			8	Insecticide spray / lotion		
			16	Mosquito coils / electric insecticide pads		
			32	Burn animal dung or other local methods		
			64	Nothing done to prevent malaria		

G8M1A	Calculate trafficlight based on the combination selected in Protection Method.		Red Green or Yellow			
G8M2	**2 USERS - Who uses mosquito nets?**	*Observe and ask.*			*	
			1	Everyone - adults and children		
			2	Only children		
			4	Only adults		
G8M3	**3 MOSQUITO NETS - Are the mosquito nets effective?**	*Observe and ask.*			*	
			1	Long lasting insecticide treated nets (LLITN)		
			2	Insecticide treated net (ITN)		
			4	Non-treated net		
			8	Good condition – no holes		
			16	Poor condition but repaired		
			32	Net with holes / not repaired		
G8M4	**4 BREEDING SITE CONTROL - Do you prevent mosquito breeding sites in any way?**	*Observe and ask.*			*	
			1	Multiple prevention methods used		
			2	Emptying pot holes and tins/ drain standing water		
			4	Cutting bush around house		
			8	Application of oils on stagnant water surfaces		
			16	Other traditional		
			32	Nothing done		
G8M5	**5 REPORTING - Have you reported any malaria cases from your household this year to the Health Centre?**	*Ask.*			*	
			1	All malaria cases reported to the Health Centre		
			2	Some malaria cases reported to Health Centre		
			4	None of the malaria cases reported to Health Centre		

**G8MX**	Has the household scored RED in any of the Malaria Protection questions?	If yes, then this household is at risk and needs improvement.	YES or NO			
**G8MY**	Has the household scored YELLOW in any of the Malaria Protection questions?	If yes, then this household has made progress but needs further improvement.	YES or NO			
**G8MZ**	RED, YELLOW or GREEN	*The household has achieved the following score for this indicator.*<br>*If Red, household is at risk and needs to improve.*<br>*If Yellow, household has made progress but needs further improvement.*<br>*If Green, congratulate household as they have achieved excellent hygiene standards for this indicator.*				
G9N1	**1 PROTEIN INTAKE - How many times in the last week have you eaten fish or meat?**	*Ask.*			*	
			1	Every day		
			2	Three times this last week		
			4	Twice this last week		
			8	Once in the last week		
			16	Never		
G9N2	**2 VEGETABLE INTAKE - How many times in the last week have you eaten vegetables?**	*Ask.*			*	
			1	Every day		
			2	Three times this last week		
			4	Twice this last week		
			8	Once in the last week		
			16	Never		
G9N3	**3 COOKED LEFTOVER STORAGE -** Where do you store your leftover cooked food?	*Observe where leftover cooked food is stored.*			*	
			1	Stored in fridge		

			2	Stored in closed cupboard		
			4	Stored in covered dish / pot		
			8	Stored in open pot / dish inside the home		
			16	Stored in open pot / outside of the home		
			32	No leftover food stored		
G9N4	**4 PLACE OF PRODUCE STORAGE -** Where do you store your produce?	*Observe where staple food is stored e.g. rice, beans, maize.*			*	
			1	All food in Model Kitchen		
			2	All inside in Model Food Store		
			4	Well stored in dedicated place		
			8	Stored anywhere		
			16	Not stored		
G9N5	5 FOOD STORE STANDARD	*Observe hygiene status of food and / or produce storage.*			*	
			1	Excellent: clean floor, shelves & closed containers		
			2	Good: shelves & closed containers		
			4	Average: closed containers		
			8	Poor: damp dirty floor, no closed containers		
			16	Very poor: dirty damp floor, flies, vermin		
**G9NX**	Has the household scored RED in any of the Nutrition questions?	If yes, then this household is at risk and needs improvement.	YES or NO			
**G9NY**	Has the household scored YELLOW in any of the Nutrition questions?	If yes, then this household has made progress but needs further improvement.	YES or NO			
**G9NZ**	RED, YELLOW or GREEN	*The household has achieved the following score for this indicator.*<br>*If Red, household is at risk and needs to improve.*<br>*If Yellow, household has made progress but needs further improvement.*<br>*If Green, congratulate household as they have achieved excellent hygiene standards for this indicator.*				
G10C1	1 PLACE OF COOKING	*Observe the place of cooking.*			*	

			1	Dedicated Model Kitchen		
			2	Outside permanent kitchen		
			4	Outside temporary kitchen		
			8	Cooking shack shared with livestock		
			16	No special place / fire made anywhere		
G10C2	2 CONSTRUCTION OF COOKING	*Observe the standard of the cooking place.*			*	
			1	Permanent		
			2	Semi permanent		
			4	Temporary		
			8	Shack		
G10C3	3 INTERIOR FACILITIES OF COOKING PLACE	*Observe the storage facility inside.*			*	
			1	Model Kitchen		
			2	Good kitchen		
			4	Upgraded shelter		
			8	Cooking shack		
			16	No storage facility		
G10C4	**4 FUEL USED FOR COOKING - What do you use for cooking?**	*Observe and ask.*			*	
			1	Electricity / Gas		
			2	Fuel efficient stove & wood		
			4	Paraffin stove		
			8	Charcoal		
			16	Firewood		
G10C5	5 LIVESTOCK CONTAMINATION	*Observe if livestock is near cooking area and / or has access to it.*			*	
			1	Household has no livestock		
			2	Livestock kept in clean stall far from cooking area		
			4	Cooking area prevents access to any livestock		
			8	Livestock tethered inside or next to cooking place		

			16	Livestock able to access cooking place		
G10C6	6 CLEANLINESS	*Observe the cleanliness of the cooking place.*			*	
			1	Excellent: well swept, no garbage		
			2	Good: quite well swept		
			4	Fair: some sweeping, only recent garbage		
			8	Poor: dirty remains of food & garbage		
			16	Very poor: no sweeping, a lot of food remains		
**G10CX**	Has the household scored RED in any of the Cooking questions?	If yes, then this household is at risk and needs improvement.	YES or NO			
**G10CY**	Has the household scored YELLOW in any of the Cooking questions?	If yes, then this household has made progress but needs further improvement.	YES or NO			
**G10CZ**	RED, YELLOW or GREEN	*The household has achieved the following score for this indicator.*<br>*If Red, household is at risk and needs to improve.*<br>*If Yellow, household has made progress but needs further improvement.*<br>*If Green, congratulate household as they have achieved excellent hygiene standards for this indicator.*				
G11CC1	**1 CLOTHING CLEANLINESS**	*Observe the state of children's clothes.*			*	
			1	Children in clean clothes		
			2	Average standard – fairly clean clothes		
			4	Children in dirty clothes		
G11CC2	**2 SEGREGATED BEDROOMS**	*Observe whether children have their own bed.*			*	
			1	Boys and girls sleep in different rooms		
			2	Boys and girls in same room but have own beds		
			4	Boys and girls share same bedding		
G11CC3	**3 ADULT SHARING**	*Observe whether children sleep in the same room as their parents.*				
			1	Children over 2 years have seperate rooms from adults		
			2	Children over 5 years have seperate rooms from adults		
			4	All children and adults sleep in same room		

G11CC4	**4 CLEANLINES OF CHILDREN'S FACES**	*Observe the faces of the children under 5 years in the household.*			*	
			1	All children with clean faces		
			2	Some children with dirty faces		
			4	All children with dirty faces		
G11CC5	**5 CHILDREN'S HEAD**	*Observe the heads of the children under 5.*			*	
			1	All children have clean hair / no lice / no ringworm		
			2	Some children have clean hair / no lice / no ringworm		
			4	All children have dirty hair and have lice / ringworm		
**G11CCX**	Has the household scored RED in any of the Child Care questions?	If yes, then this household is at risk and needs improvement.	YES or NO			
**G11CCY**	Has the household scored YELLOW in any of the Child Care questions?	If yes, then this household has made progress but needs further improvement.	YES or NO			
**G11CCZ**	RED, YELLOW or GREEN	*The household has achieved the following score for this indicator.*<br>*If Red, household is at risk and needs to improve.*<br>*If Yellow, household has made progress but needs further improvement.*<br>*If Green, congratulate household as they have achieved excellent hygiene standards for this indicator.*				

End of Survey
