# Supplementary material for: The value of monitoring data in a process evaluation of hygiene behaviour change in Community Health Clubs to explain findings from a cluster-randomised controlled trial in Rwanda
Source: BMC Public Health. 2020 Jan 23;20:98. doi: 10.1186/s12889-019-7991-7 (PMC6979057; doi:10.1186/s12889-019-7991-7)
Supplement: Supplementary file 2 — Additional file 2. Indicators of Community Response. [file 12889_2019_7991_MOESM2_ESM.docx]

***Indicators of Community Response***

|  |  |  | | **# CHCs** | **% CHCS** |
| --- | --- | --- | --- | --- | --- |
| **Indicator 1**: | **>70 members per CHC** | | |  |  |
|  | **Total members in 50 CHCs** | | | **4056** |  |
| High | 70 -100 members registered | | | 36 | 72% |
| Good | 50-69 members registered | | | 13 | 26% |
| Average | 30-49 members registered | | | 1 | 2% |
| Low | <30 members registered | | | 0 | - |
| **Indicator 2** | **80 % coverage of households** | | |  |  |
|  | **Households with a member in a CHC / % of all CHCs** | |  | **4056** | **58.4%** |
| High | >80% of households with a member in a CHC | |  | 11 | 22% |
| Good | 60-79% of households with a member in a CHC | |  | 17 | 33% |
| Average | 40 - 59% of households with a member in a CHC | |  | 16 | 31% |
| Low | <40% of households with a member in a CHC | |  | 7 | 14% |
| **Indicator 3** | **50% Average attendance per session per CHC** | | |  |  |
|  | **Total # attendances in all CHCs** |  | | **2035** |  |
|  | **Mean attendance** |  | |  | **41%** |
| High | > 75% attendance |  | | 4 | 8% |
| Good | 50%-74% attendance |  | | 11 | 22% |
| Average | 30-49% attendance |  | | 20 | 40% |
| Low | <30% attendance |  | | 15 | 30% |
| **Indicator 4:** | **50 % CHC members graduating** | | | |  |
|  | **# of members graduating in all CHC (%) 2470** | | | | **60.8%** |
| High | 80% -100% of members |  | | 6 | 12% |
| Good | 50%-79% of members |  | | 19 | 38% |
| Average | 30% - 49% of members |  | | 15 | 30% |
| Low | <30% of members |  | | 10 | 20% |
